# The Surgical Treatment of Neovascular Glaucoma with Ex-PRESS P-50 Miniature Glaucoma Shunt

**DOI:** 10.5005/jp-journals-10008-1102

**Published:** 2012-10-16

**Authors:** Dewang Angmo, Amit Sobti, Anita Panda

**Affiliations:** 1 Department of Glaucoma Services, Dr RP Centre for Ophthalmic Sciences, All India Institute of Medical Sciences, New Delhi, India; 2 Department of Glaucoma Services, Dr RP Centre for Ophthalmic Sciences, All India Institute of Medical Sciences, New Delhi, India; 3 Department of Glaucoma Services, Dr RP Centre for Ophthalmic Sciences, All India Institute of Medical Sciences, New Delhi, India

**Keywords:** Neovascular glaucoma, Glaucoma shunts, Ex-PRESS mini glaucoma shunt.

## Abstract

Surgical treatment for neovascular glaucoma has evolved from cyclodestructive procedures to full-thickness filtration surgery, to trabeculectomy with antifibrosis drugs and glaucoma drainage implant surgery. The choice of a surgical approach may be influenced by several factors, including the stage of the disease. Many surgeons favor drainage implants when the disease is more advanced or when severe inflammation is present, which would be associated with a poorer prognosis if trabeculectomy were chosen. New devices are being developed to allow surgeons increased control with more predictable postoperative results. One such device, the Ex-PRESS™ mini glaucoma shunt, has undergone changes in design and method of insertion making it more appealing for use in patients requiring IOP-lowering surgery. This report highlights the use of Ex-PRESS mini glaucoma shunt in neovascular glaucoma, surgical technique and summarize pertinent literature on the role of this device in glaucoma surgery.

## CASE REPORT

A 45-year-old male presented to outpatient department of our hospital with history of pain and diminution of vision in both eyes L>R. He is a known case of diabetes mellitus since 10 years (on insulin and oral hypoglycemic agents). He was diagnosed as OD: Lasered proliferative diabetic retinopathy with neovas-cular glaucoma; OS: Lasered proliferative diabetic retinopathy with CSME with a baseline IOP of 60 mm Hg in the right eye and 12 mm Hg in the left eye. The visual acuity in the right eye was no PL and in the left eye was 2/60, PR accurate with a near total cupping in the right eye and 0.4:1, NRR temporal pallor. Urgent control of IOP with IV mannitol following which patient was prescribed eyedrop combigan BD and eyedrop dorzox TDS and was advised anterior retinal cryopexy in the right eye and pan retinal photocoagulation in the left eye. These procedures were performed in the subsequent week and the PRP was completed in 4 weeks duration. On follow-up at 2 and 4 weeks the IOP was OD: 38, 40 mm Hg; OS: 14,16 mm Hg. The patient failed to follow-up and presented 6 months later due to uncontrolled blood sugar (diabetic ketoacidosis) for which he was hospitalized. On presentation right eye no PL, IOP-50 mm Hg, closed angles and left eye PL+, PR inaccurate in three quadrants, IOP-48 mm Hg, closed angles. Patient underwent left eye glaucoma shunt surgery (Ex-PRESS mini-shunt) with MMC 0.04% under local anesthesia.

### Description of Device

The Ex-PRESS glaucoma filtration device (Alcon Inc, Fort Worth, TX) is a small stainless steel, nonvalved flow-restricting, MRI compatible device^1,8^ designed to lower intraocular pressure in glaucomatous eyes by diverting aqueous humor through the implant from the anterior chamber to an intrascleral space―the bleb.^2-5^

**Figs 1A and B:**
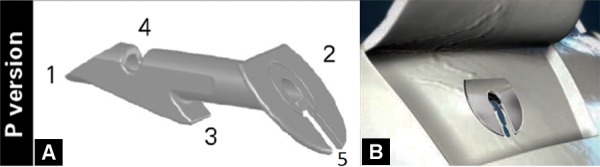
(A) P-50 version the Ex-PRESS glaucoma filtration device, (B) a notch to facilitate posterior flow of aqueous

It consists of a 2 to 3 mm long tube with 400 micron external tube diameter and 50 micron internal tube diameter, which connects the anterior chamber to the intrascleral space. Despite its miniature size, the Ex-PRESS glaucoma filtration device features several major structural elements^4^ (Figs 1A and B):

1. A cannula for draining aqueous humor from the anterior chamber to the intrascleral space.
2. A plate to prevent excessive penetration.
3. A spur to prevent extrusion of the Ex-PRESS^®^ glaucoma filtration device from the eye.
4. Reserve orifices near the distal end, which constitute an alternative conduit for aqueous humor drainage in case of occlusion of the primary (axial) opening of the cannula by the iris.
5. A notch to facilitate posterior flow in the P-50 model.

The Ex-PRESS glaucoma filtration device is preloaded on a specially designed disposable introducer, the Ex-PRESS delivery system (EDS-Fig. 2C). The EDS is an inserter designed to maintain the correct orientation of the Ex-PRESS glaucoma filtration device throughout the implantation procedure. It allows the surgeon better control of the device as it is released. It enables easy insertion for either right or left handed physicians, using only one finger for simple, consistent device release and is intended for single use only.

**Fig. 2A:**
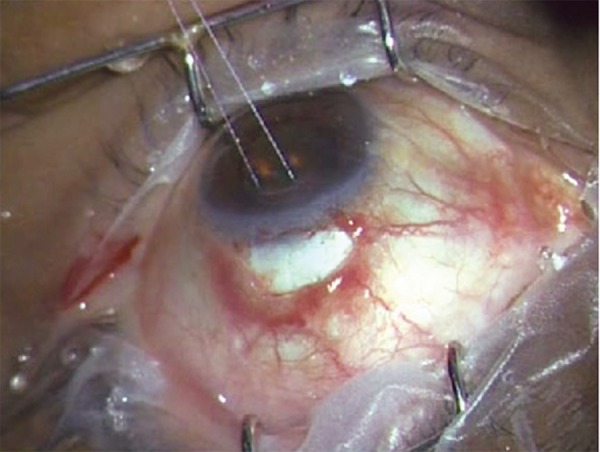
Standard fornix-based conjunctival incision with corneal traction suture

**Fig. 2B:**
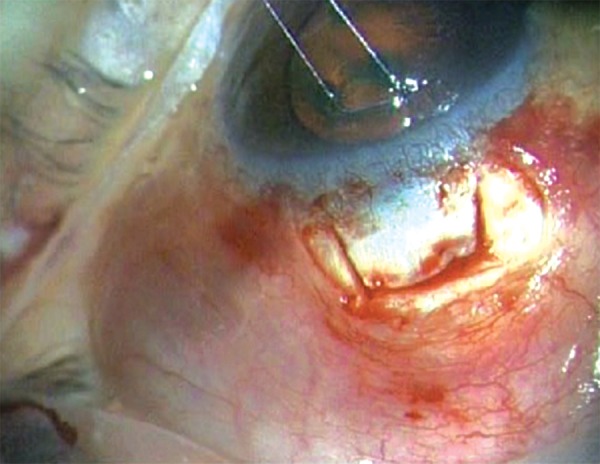
Scleral flap 4 × 4 mm made

**Fig. 2C:**
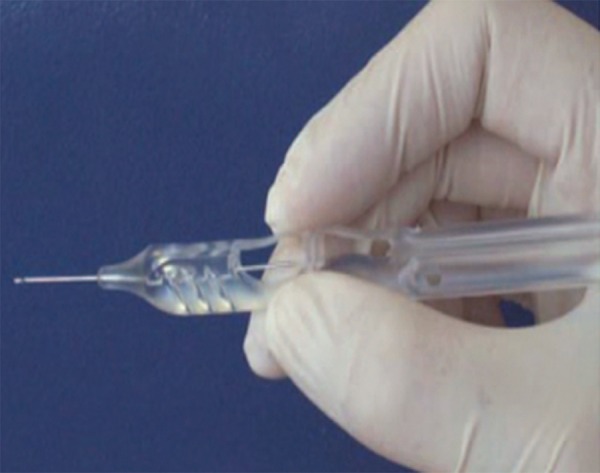
The Ex-PRESS delivery system (EDS)―A specially designed preloaded disposable introducer for the Ex-PRESS mini glaucoma shunt

**Fig. 2D:**
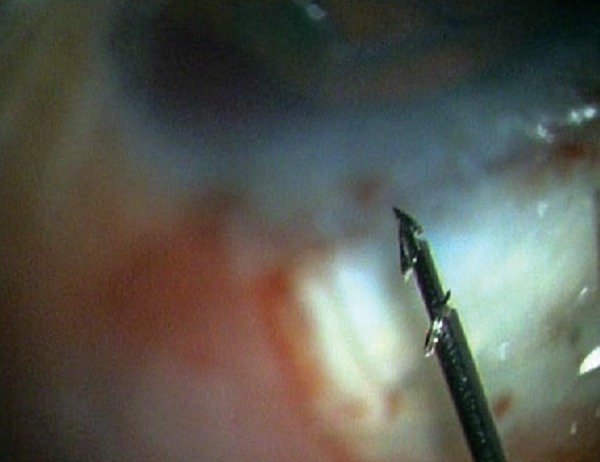
High magnification view of the tip of the EDS showing the shunt

**Figs 2E and F:**
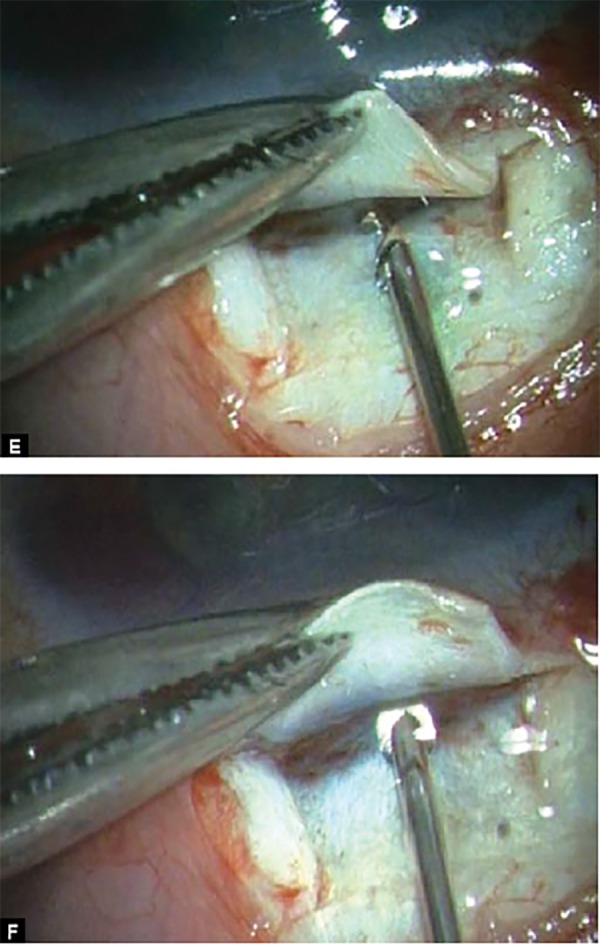
The shunt is inserted into the anterior chamber through the center of the ‘blue line’ at an angle parallel to the iris plane via a preplaced track made with 26G needle

The following versions of the Ex-PRESS glaucoma filtration device are commercially available: R-50, P-50 and P-200.

### Surgical Procedure

1. A standard fornix-based (Fig. 2A) conjunctival incision is made to gain exposure to the scleral bed adjacent to the limbus.
2. Gentle cautery is performed in this area.
3. A scleral flap (Fig. 2B) is created in a similar manner performed with a standard trabeculectomy (4 × 4 mm). Care is taken to dissect the flap up to clear cornea.
4. Mitomycin-C 0.04% soaked pledgets is applied subcon-junctivally for 2 minutes and subsclerally for 1minute after coating the cornea with viscoelastic.
5. A temporal paracentesis is created through the cornea.
6. The scleral flap is lifted and care is taken to identify the center of the ‘blue line’ adjacent to clear cornea which corresponds to the location of the trabecular meshwork. A 26 gauge needle is inserted into the anterior chamber through the center of the ‘blue line’ at an angle parallel to the iris plane. The needle is removed ensuring no lateral movement of the needle as this will cause aqueous to flow around the implant.
7. The Ex-PRESS shunt is preloaded on an injector.^3,18^ Fitted into the lumen of the shunt is a metal rod that is attached to the end of the injector (Figs 2C and D).
8. The shunt is then placed in the anterior chamber through the ostium created with the needle. The angle of entry with the shunt is the same as the angle used to make the ostium (Figs 2E and F). The shunt is inserted all the way into the wound making the plate flush with the scleral bed. In a similar fashion as a standard punctual plug inserter, the injector has an area on the shaft that is then depressed which retracts the metal rod in the lumen of the shunt. This allows the injector to be free from the lumen of the shunt.
9. The functioning of Ex-PRESS shunt is ensured by applying few drops of methylene blue dye 0.06% over the ostium and immediate disappearance of the dye noted as aqueous is shunted from anterior chamber (Fig. 2G).
10. The scleral flap is then sutured in place using a 10-0 nylon suture with a spatulated needle (Fig. 2H). One to three sutures are typically required depending on the flow which can be tested by inflating the anterior chamber with balanced salt solution with a 27 or 30 gauge cannula through the temporal paracentesis.
11. The conjunctiva is then meticulously closed with 8-0 vicryl in a running fashion. Ex-PRESS shunt *in situ* with a well formed anterior chamber and a raised filtering bleb postoperatively (Fig. 2I).

**Fig. 2G:**
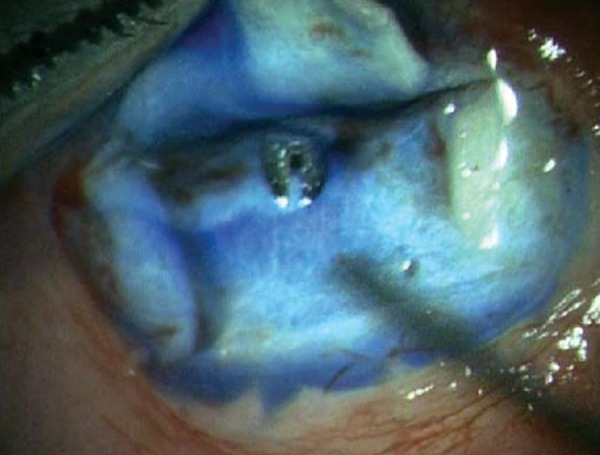
Ensuring functioning of Ex-PRESS shunt by applying few drops of methylene blue dye 0.06% over the ostium and noting immediate disappearance of the dye as aqueous is shunted from anterior chamber

**Fig. 2H:**
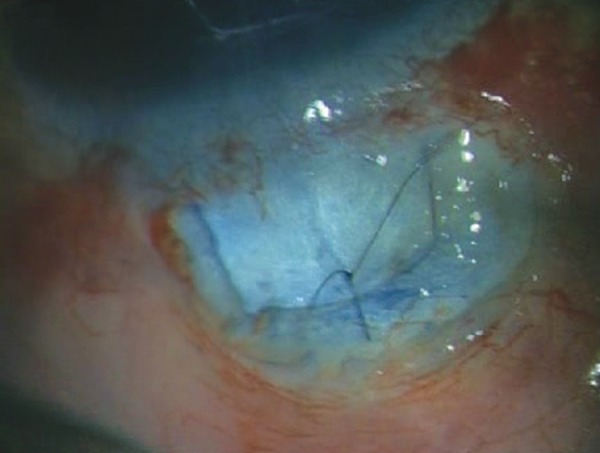
Scleral flap is sutured in place using a 10-0 nylon suture

**Fig. 2I:**
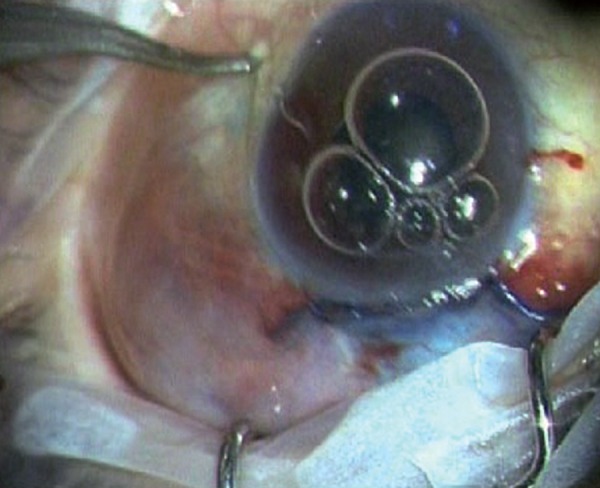
Ex-PRESS shunt *in situ* with a well-formed anterior chamber and a raised filtering bleb

## DISCUSSION

Aqueous shunts are preferred by many for NVG not responding to PRP and maximal medications. Advantages of shunts include no necessity for an iridectomy during anterior chamber installation, decreasing the risk of bleeding. In any case, a shunt is likely to better tolerate intraoperative and postoperative bleeding or massive fibrin reaction than trabeculectomy with an increased likelihood of continued function.^6^

The Ex-PRESS mini glaucoma shunt was developed as a less invasive surgical procedure^2,5^ compared with conventional trabeculectomy and has two distinct advantages, including a consistently sized scleral fistula and no need for iridectomy. It is inserted under a scleral flap to shunt aqueous humor from the anterior chamber to the subconjunctival space using a filtration bleb.^4^

Several studies have reported on the efficacy of Ex-PRESS,^5,7,9,10^ but only one randomized the surgical procedures.^9^ While the efficacy results consistently demonstrated significant intraocular pressure reductions, they differed in the frequency of complications, with more such events occurring with earlier surgical techniques in which the Ex-PRESS device was implanted under a conjunctival flap.^5,9,10^ These complications have been well described in the literature.^3-5,9-17^ Endophthalmitis has also been associated with an exposed implant.^12^ To avoid such complications, Dahan and Carmichael proposed implanting the device under a scleral flap.^2^ This technique has almost completely eliminated the erosion complication and has been demonstrated to have a lower rate of hypotony than trabeculectomy.^3^

Kanner et al^19^ published a comparative consecutive case series of 345 patients, 231 eyes treated with Ex-PRESS implant under scleral flap alone and 114 eyes treated with Ex-PRESS implant under scleral flap combined with phacoemulsification. At 3 years after surgery, surgical success was 94.8% and 95.6% in the Ex-PRESS and combined groups respectively. The change from baseline IOP was significantly greater after Ex-PRESS implant alone compared with combined surgery. Of interest, the most common device-related complication was obstruction of the tube with fibrin in patients (1.7%), which was treated successfully with Nd:YAG laser.

It has been demonstrated by a previous paper by Maris et al that there is a lower incidence of hypotony with the Ex-PRESS compared with trabeculectomy.^3^ Maris et al demonstrated a 32% hypotony rate in the trabeculectomy group and 4% in the Ex-PRESS group.^3^ In Kanner et al, there was 15.6% with hypotony (IOP <5 mm Hg) in the first week in the Ex-PRESS alone group, and 7.9% with hypotony in the first week in the combined group. All of these instances of hypotony during the early postoperative period resolved spontaneously. None of the eyes developed flat anterior chamber with lens-cornea touch.^19^

It is postulated that the lower rate of hypotony is from the resistance to flow that is offered by the 50 micron lumen of the shunt. Whereas, with trabeculectomy, the smallest scleral punch that is manufactured is approximately 750 microns and that is only if one punch alone is used to make the incision. It is clear that scleral sutures offer much of the resistance to flow with either trabeculectomy or the Ex-PRESS shunt; however, with the latter it is likely that both the small lumen of the implant and the suture tensioning offer resistance.
